# Genotype-dependent lifespan effects in peptone deprived *Caenorhabditis elegans*

**DOI:** 10.1038/srep16259

**Published:** 2015-11-05

**Authors:** Jana J. Stastna, L. Basten Snoek, Jan E. Kammenga, Simon C. Harvey

**Affiliations:** 1 Biomolecular Research Group, Canterbury Christ Church University, Canterbury, CT1 1QU, UK; 2 Laboratory of Nematology, Wageningen University, 6708 PB Wageningen, The Netherlands

## Abstract

Dietary restriction appears to act as a general non-genetic mechanism that can robustly prolong lifespan. There have however been reports in many systems of cases where restricted food intake either shortens, or does not affect, lifespan. Here we analyze lifespan and the effect of food restriction via deprived peptone levels on lifespan in wild isolates and introgression lines (ILs) of the nematode *Caenorhabditis elegans.* These analyses identify genetic variation in lifespan, in the effect of this variation in diet on lifespan and also in the likelihood of maternal, matricidal, hatching. Importantly, in the wild isolates and the ILs, we identify genotypes in which peptone deprivation mediated dietary restriction reduces lifespan. We also identify, in recombinant inbred lines, a locus that affects maternal hatching, a phenotype closely linked to dietary restriction in *C. elegans*. These results indicate that peptone deprivation mediated dietary restriction affects lifespan in *C. elegans* in a genotype-dependent manner, reducing lifespan in some genotypes. This may operate by a mechanism similar to dietary restriction.

Ageing is a universal phenomenon during which progressive changes ultimately lead to death. Lifespan can be affected by many factors, one being dietary restriction (DR). DR, or caloric restriction, is a reduction in caloric intake without malnutrition. This non-genetic intervention can robustly extend lifespan in variety of species from yeast to mice (e.g.^1^,^2^,^3^,^4^, for review see^5^). In a number of model species, DR has also been shown to delay the onset or severity of age-related traits that are associated with diseases such as diabetes, dementia and cardiovascular diseases^4^,^5^,^6^,^7^. Studies in primates also suggest that DR can improve health in rhesus monkeys^8^,^9^, although the effect on lifespan is less clear, with one study reporting a reduction in mortality^9^, while a second observed an improved health span and no effect on lifespan^8^. Whilst the effect of DR on human lifespan are still unknown, the accumulating data indicates that DR without malnutrition improves markers of aging (for review see^10^). More studies are needed to allow for better understanding of the molecular processes that are likely to play part in individual genotype-dependent responses to DR in humans.

Despite the general case for DR prolonging lifespan, examples exist in yeast^11^, worms^12^, fruit flies^13^, house flies^14^, rats^15^ and mice^16^,^17^,^18^ where DR either has no effect or even reduces lifespan. Some of this variation is a consequence of different methodologies and of the severity of the DR. For example, Mediterranean fruit flies showed no increase in longevity with moderate DR and dramatically increased mortality once the level of DR reached 50%^13^. Genetic background is also an important factor. For example, no effect of DR was detected in freshly caught wild mice maintained on a regime that prolonged the lifespan of lab adapted animals^17^. Similarly, changes in lifespan in response to DR were variable between recombinant inbred mouse strains^18^, with DR shortening lifespan in more of the strains than which it increased lifespan. Variable responses to DR have also been observed in a genetically heterogeneous population of budding yeast (*Saccharomyces cerevisiae*), with the effect of DR varying from a 103% increase to a 79% decrease among strains^11^.

Invertebrate species provide an excellent model for ageing research due to their relatively short lifespan and experimental tractability. One such model system is the nematode *Caenorhabditis elegans*. The effects of DR on lifespan in *C. elegans* have been investigated via several methods including the use of mutants (e.g. *eat-2*) deficient in pharyngeal pumping^19^, axenic culture^20^, bacterial dilution in liquid cultures^21^, dilution or total withdrawal of peptone from the agarose plates^22^, the use of UV-killed bacteria^23^ and total starvation^3^,^24^. All of these experimental manipulations have been successful in prolonging lifespan^25^. In *C. elegans*, the longevity response to DR is actively regulated through independent and overlapping pathways. These pathways are evolutionary conserved and include; target of Rapamycin (TOR) for chronic food limitation (strong-effect *eat-2* mutants and starvation treatments)^26^, AMP-activated protein kinase (AMPK) (bacterial dilution on plates starting in middle age and peptone dilution)^23^, sirtuins (mild DR or weak-effect *eat-2* mutants)^27^ and insulin/insulin-like growth factor (IGF-1) signaling (intermittent feeding)^28^.

In addition to the effects on lifespan, *C. elegans* responds to many poor food conditions, such as DR, by increasing the rate of maternal hatching, a situation where fertilized eggs hatch inside the reproductive tract and the resulting progeny consume the mother. Maternal hatching in *C. elegans* is also associated with ageing, with the rate increasing with age over the reproductive period. Increased maternal hatching in response to diet may therefore represent the effects of stress or damage, or may be an adaptive parental response to starvation^29^,^30^. Many ageing and most DR studies in *C. elegans* have avoided maternal hatching by assaying worms in the presence of 5-fluorodeoxyuridine (FUdR), a drug that inhibits cell division and prevents eggs from hatching. This is however potentially problematic when considering variation in the response to DR, as FUDR has been shown to affect lifespan in a genotype-dependent manner^31^,^32^ and to interact with the stress response^33^. The genetics of variation in maternal hatching is also poorly understood and although a small number of quantitative trait loci (QTL) affecting maternal hatching have been identified^34^, it is not known if these are related to variation in lifespan.

There is extensive genetic variation between wild isolates of *C. elegans*^35^,^36^,^37^, but these have only sparsely been studied and nearly all *C. elegans* research is carried out on the N2 (Bristol) strain and its derived mutants. Natural variation in *C. elegans* lifespan has however been analyzed, principally using recombinant inbred lines (RILs) and subsequent QTL mapping (for review see^38^). More recent work using introgression lines (ILs) derived from wild-types N2 and CB4856 has identified novel loci underlying natural variation in two age-related traits (pharyngeal pumping and lifespan)^39^ and age related expression QTLs^40^. It is however unknown if these QTLs also affect the response to DR. The majority of analyses of DR in *C. elegans* have been undertaken in the N2 genetic background, with one study analysing five wild isolates of *C. elegans* and two of *C. remanei*^12^ and identifying variation in response to DR by bacterial dilution, a method that results in maximal lifespan extension from DR in *C. elegans*^3^,^24^. In this study, DR treatment extended lifespan for all *C. elegans* isolates and mean lifespan was extended for one isolate of *C. remanei*^12^. To address these issues, here we have a) analyzed the effect of peptone deprivation mediated DR on lifespan and maternal hatching in ILs; b) mapped variation in maternal hatching in N2/CB4856 RILs and c) compared these data to the effects of peptone deprivation mediated DR on wild isolates. We show that maternal hatching is a genetically variable phenotype and linked loci can be found by QTL analysis. We also conclude that the effect of peptone deprivation mediated DR on lifespan is polygenic, does not exclusively prolong lifespan and depends on the genetic background.

## Results

### Variable effects of dietary restriction on introgression lines

To test if the genetic background affects the lifespan effects of peptone deprivation mediated DR we tested five ILs, each containing an introgressed portion of the CB4856 genome in an N2 genetic background that had previously been shown to affect lifespan^39^. Overall, peptone deprived worms increased mean lifespan (p = 0.005) in the ILs tested (Table 1; Supplementary Dataset 1). However, large differences between the genotypes were observed (p = 0.007). In N2 and two ILs (ewIR01 and ewIR18) peptone deprivation prolonged mean lifespan. However, peptone deprivation had no effect on lifespan in two ILs (ewIR21 and ewIR51) and reduced lifespan in another (ewIR40). This indicates that, in the ILs tested, the effects of peptone deprivation are dependent on genotype. These data also support three previously identified^39^ lifespan QTLs in lines ewlR01, ewIR21 and ewIR51 (comparisons of ILs against N2, p < 0.05) (Table 1).

Maternal hatching did also differ among these ILs, with the genotypes for which peptone deprivation prolonged lifespan (N2, ewIR01 and ewIR18) showing limited maternal hatching (Table 1). Other genotypes (ewIR21, ewIR40 and ewIR51) showed increased maternal hatching under both peptone deprived and control (NGM) conditions. As individuals that died via maternal hatching were not included in the analysis of lifespan data, the lack of lifespan extension in response to peptone deprivation in these ILs is not a result of increased maternal hatching. QTLs affecting maternal hatching have previously been identified in ewIR21 and ewIR51^34^, this analysis therefore identifies an additional QTL at the introgression of ewIR40. Moreover, the analysis shows that these QTLs also cause increased maternal hatching in response to peptone deprived DR. As we detected multiple shared loci involved in lifespan, the effect of peptone deprivation on lifespan and maternal hatching, these results suggest a link between the lifespan prolonging effect of peptone deprivation mediated DR and maternal hatching.

### Identification of quantitative trait loci associated with maternal hatching

**Figure 1:**
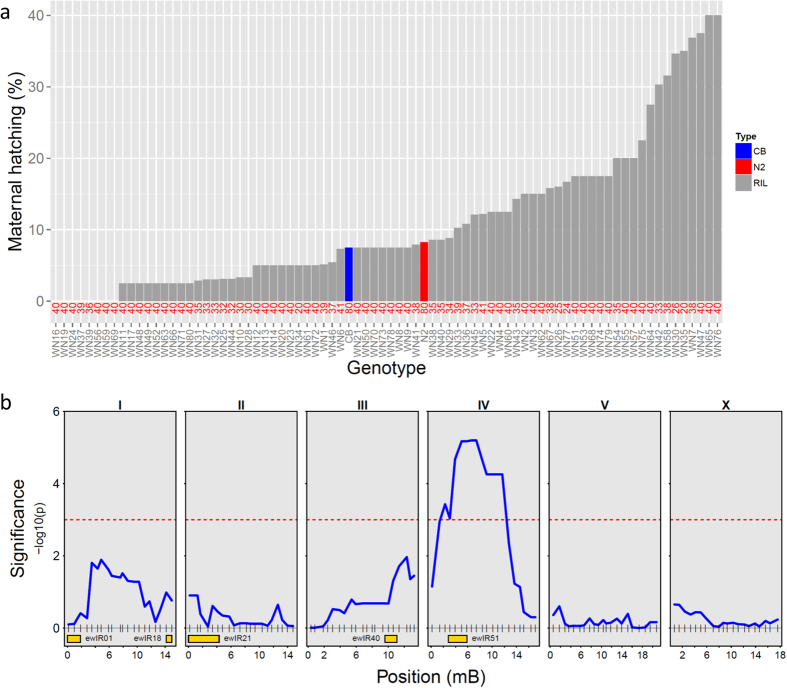
Maternal hatching in N2/CB4856 RILs. (**a**) Percentage maternal hatching in N2/CB4856 RILs, CB4856 (blue) and N2 (red), this identifies transgressive segregation. Total animals per line is indicated by the red numbers on the x-axis. (**b**) QTL profile for the percentage of worms that died by maternal hatching. Significance (−log10(p), blue lines) plotted against the marker position in mega base pairs (grey vertical lines on the 0 line). Chromosome names are indicated above the panels. Threshold (0.05; 1000 permutations) is shown by the dotted red line.

**Figure 2:**
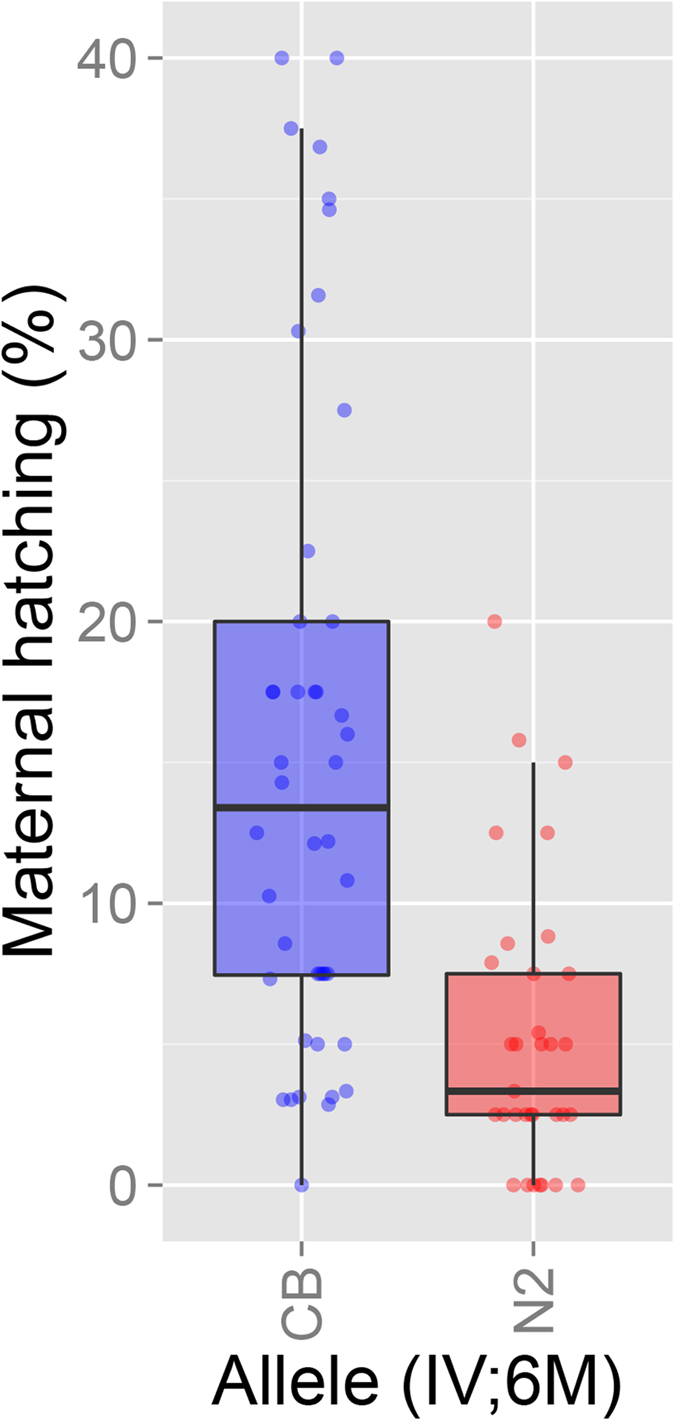
Allelic effect on maternal hatching. The maternal hatching rates of the RILs grouped by their allele on chromosome IV at ~6M basepairs, CB4856 (blue) and N2 (red).

To test if variation in maternal hatching can be linked to more loci we used RILs produced from crosses between N2 and CB4856^41^. We found extensive variation between the RILs and transgression in maternal hatching (Fig. 1a). One major QTL for maternal hatching rate could be found roughly at the position of the CB4856 introgression in IL ewIR51 (Fig. 1b; left arm CHR. IV), with this QTL explaining 22.8% of the trait variation. Taking marker 66 (at chromosome IV ~6M) as the peak of this maternal hatching QTL, we found an effect of 5.2%, such that the maternal hatching rate was 10.4% higher for the CB4856 allele than the N2 allele (Fig. 2). This is confirmed by the 10% maternal hatching of ewIR51. The two other loci found in ewIR21 and ewIR40 (Table 1) were not detected in the RILs (Fig. 1b) even though they had a larger effect and increased maternal hatching to almost double of that of ewIR51. This suggests an interaction of these two loci with the genetic background. Maternal hatching is therefore likely to be a complex polygenic trait and genetically balanced in the parental lines.

### Natural variation among wild isolates of C. elegans

**Figure 3:**
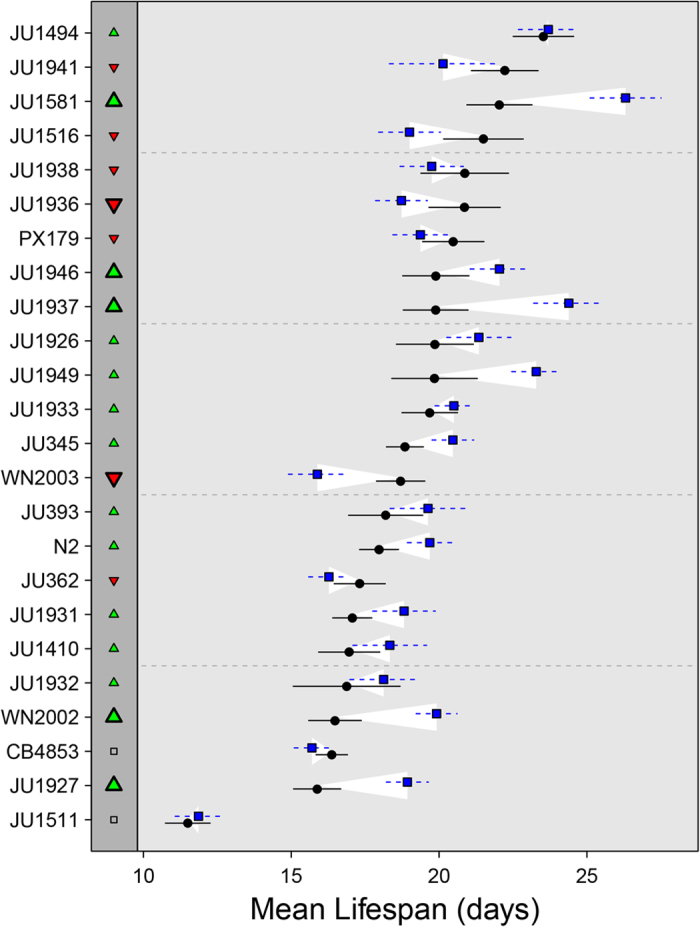
Lifespan and the effect of peptone deprived dietary restriction in wild isolates. Mean lifespan, ±SE, under (dots) normal (NGM) and (squares) peptone deprived conditions. The white marked regions denote the effect of DR on mean lifespan. The effect of DR on lifespan is also indicted by the triangles on the left, point upwards indicates a positive effect of DR and point downwards a negative effect of DR. Large triangles indicate a significant difference between NGM and DR.

**Figure 4:**
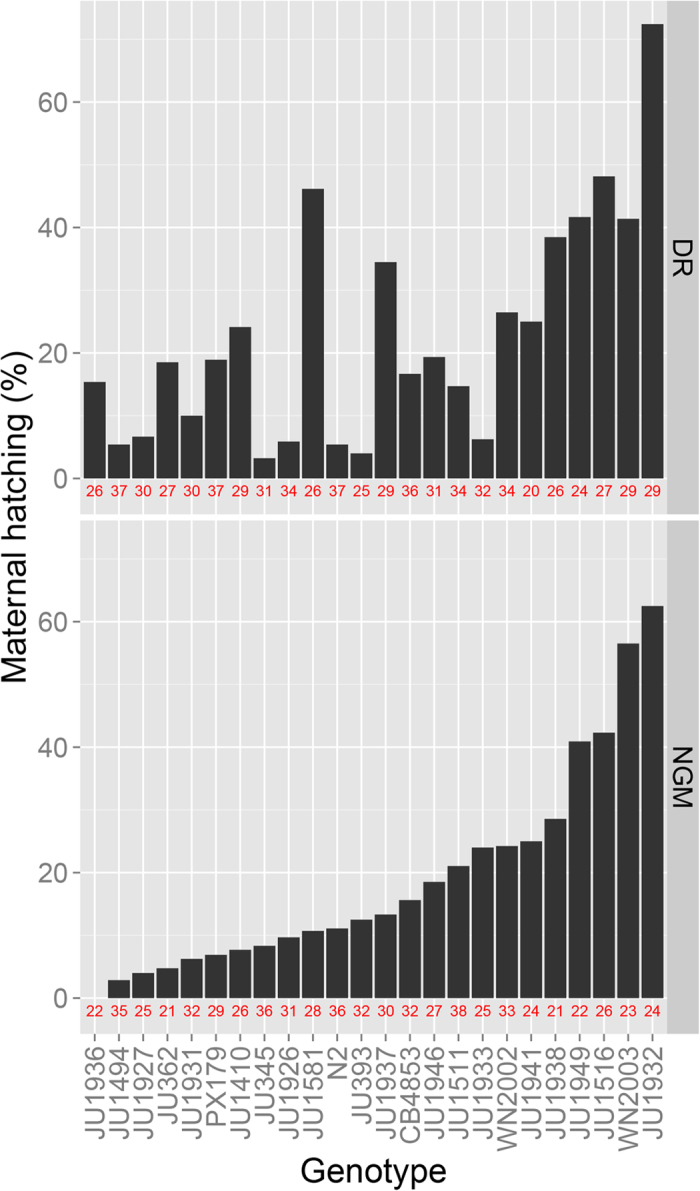
Maternal hatching rate of the wild-isolates on peptone deprived DR (top panel) and NGM (lower panel). Wild isolates were ordered by their maternal hatching rate on NGM. Total number of animals tested for each wild isolate and condition shown by the red numbers on the x-axis.

To investigate if lifespan, maternal hatching and the effect of peptone deprivation could also be under balancing selection in nature, we studied these traits in 23 wild isolates and N2 (Figs 3 and 4; Supplementary Dataset 2). Remarkably, peptone deprivation reduced lifespan in about a third of the wild isolates in comparison to N2, with the effect ranging from an increase in mean lifespan in JU1937 of 4.5 days to a decrease of 2.8 days in WN2003 (Fig. 3). From these results we can conclude that there is genetic variation for peptone deprived mediated DR within *C. elegans* and that it does not extend lifespan in all genotypes. We also found large variation in maternal hatching between the wild isolates, however, unlike in the ILs, this does not appear to be associated with lifespan or the effect of peptone deprived mediated DR on lifespan. These results are therefore likely to reflect genotype-dependent responses to the environment.

## Discussion

In this study, we have focused on natural variation in lifespan, the response to peptone deprived dietary restriction and on maternal hatching in *C. elegans.* In ILs we identified 3 QTLs affecting lifespan, 3 affecting the response to peptone deprivation and 3 affecting maternal hatching. This analysis confirms 3 (ewIR01, ewIR21, ewIR51) of 5 previously identified lifespan QTLs in this panel of ILs^39^. Differences between the studies are likely to be due to either lab-to-lab differences or the small population sizes used in previous analyses^39^. Assembly of the CB4856 genome indicates that there are a number of regions of exceptionally high divergence between N2 and CB4856^42^. Comparison of the location of these regions to the limits of the introgressions in the ILs analyzed here indicates that three of the ILs (ewIR01, ewIR21, ewIR51) contain such highly diverged regions, but that ewlR18 and ewlR40 do not. Given this, the effects of these diverged regions on lifespan may therefore warrant further analysis.

We also find the ILs respond differently to peptone deprivation, with peptone deprivation extending the lifespan of two ILs, having no effect on two other ILs and reducing the lifespan of a fifth IL (Table 1). Critically, we find that peptone deprivation affects lifespan in a genotype specific manner, making it likely that even mild peptone deprived mediated DR will affect the detection of some lifespan QTLs. A DR regimen of total peptone withdrawal from NGM plates was chosen for its mild DR effect^22^. Under this condition, the *Escherichia coli* OP50 does not grow on the plates but is not killed. Whilst the molecular mechanisms of dietary interventions are still poorly understood, it is known that various DR methods operate via independent and overlapping pathways^25^. For example the low-energy-sensing AMPK is required for lifespan extension in bacterial and peptone dilution on NGM plates^25^.

The three ILs in which peptone deprivation failed to prolong lifespan showed increased maternal hatching in both feeding regimes, with a significant increase observed in response to peptone deprivation. Although the increased maternal hatching does not directly explain the lack of a response to peptone deprivation, the overlap between the QTLs suggests that there is a relationship between the response to peptone deprivation and the increased maternal hatching. It is likely that this is a consequence of an underlying factor affecting both traits. One possibility is that increased maternal hatching is caused by a maternal response to the peptone deprived environment which in turn leads to sacrificing themselves to allow progeny development as proposed by^30^. This scenario seems unlikely in this case given the variation between lines and the mild form of DR used. An alternative possibility is that the differences are due to genetic incompatibilities between CB4856 alleles and the N2 background^34^, with these incompatibilities affecting both maternal hatching and the response to DR.

Comparison of maternal hatching in the ILs and the RILs (Table 1 vs Fig. 1b) indicates that the major QTL found in the RILs co-locates with one of the QTLs found in the ILs (ewIR51). The two other QTLs in the ILs were not found in the RILs. This might be due to differences in experimental settings or the more complex background of the RILs compared to the ILs. It could also be due to alleles being more balanced in the RILs preventing the maternal hatching phenotype in many combinations. This observation mirrors the observed pattern of trait variation identified in a previous comparison of ILs and RILs in *C. elegans*^34^, with analysis of ILs revealing more loci affecting the trait than are seen in RILs. This may imply that the effects observed, particularly the observed reduction in lifespan in response to peptone deprivation observed in ewIR40, are a consequence of incompatibilities between CB4856 alleles and the N2 genetic background.

Arguing against this explanation is the observed variation in lifespan, in peptone deprivation mediated DR and in maternal hatching observed in the wild isolates from various locations in France and The Netherlands. The genetic and gene expression differences between these wild isolates are characterized by gene-environment signatures^36^, and it is therefore reasonable to expect that different isolates will display a differential response to peptone deprivation treatment. Peptone deprivation had a life prolonging effect on most wild isolates. Thus, the overall effect of peptone deprivation is an extended lifespan. However, seven wild isolates were shorter lived under peptone deprivation compared to control conditions and an additional three isolates showed negligible effect of DR on mean lifespan. Together with the results of the IL analysis, this shows that peptone deprivation is not a universal method of lifespan extension in genotypes obtained from specific crosses as well as genotypes freshly taken from their natural habitat. This mirrors findings in mice and rats, where extensive variation between genotypes has been observed. In these rodent systems, a possible explanation for this variation relates to lab adaptation and the effects of inbreeding^17^,^18^ (also see^43^, for discussion of this variation). Given that both wild isolates and lab isolates of *C. elegans* are highly inbred, our results are not likely to represent a lab adaptation, but could be explained as genetic plasticity in response to diminished resources rather than that of true caloric restriction. As previously stated the peptone dilution in NGM plates is relatively mild form of DR in *C. elegans*^22^, with other DR regimens producing greater extensions of lifespan in an N2 genetic background^19^,^20^,^21^,^23^,^24^. Given that various DR regimes extend lifespan via independent and overlapping genetics pathways^25^, it would therefore be of great interest to determine if variation between genotypes depended on the method of DR.

Most studies in *C. elegans* have been undertaken using one canonical strain, Bristol (N2) as a reference genotype. The N2 strain was maintained in continuous culture for about 13 years prior to freezing thus subjected to very different condition to that of in the wild^44^,^45^. It is therefore likely to be extensively adapted to laboratory conditions^44^,^46^ and the genes affecting longevity and the DR response could have been affected by this. Recently, more thought has been given in *C. elegans* research to comparisons of N2 with natural wild isolates^35^,^36^,^37^,^45^,^47^. Knowing that genetic variation can significantly influence response to peptone deprivation mediated DR, a closer look at the freshly derived-wild isolates is therefore needed to obtain a more realistic idea about the effects of DR and to find the natural polymorphic alleles involved in the response to DR. This also highlights the need for studies in general to be carried out in various genetic backgrounds.

## Methods

### *C. elegans* isolates

Worms used were N2 (Bristol), the N2/CB4856 ILs ewIR1, ewIR18, ewIR21, ewIR40 and ewIR51^39^, N2/CB4856 RILs^40^,^41^,^48^,^49^,^50^,^51^, and wild isolates^36^ (donated by M.A. Felix to JK). The selected ILs contain five of six previously identified QTLs with potential life-shortening effects of the CB4856 allele^39^. An additional lifespan QTL identified in these ILs^39^ was not retested here as the QTL region contains *npr-1*. As variation between N2 and CB4856 affects many traits in *C. elegans*, with many of these effects mediated by the effects of behavior on food availability^52^,^53^, this QTL was not analyzed in this study. All lines were maintained at 20 °C on standard nematode growth medium (NGM) with *Escherichia coli* OP50 as a food source^54^.

### Lifespan assays ILs and wild isolates

Worms were grown *en masse* to adulthood and eggs were collected from sodium hypochlorite treated gravid adults^54^. These eggs were then maintained for 24 hours without food at 20 °C, with synchronized L1s then transferred to fresh 35 mm plates (n = 40 per treatment per genotype, with 1 worm per plate for the ILs and 5 worms per plate for the wild isolates). Control, *ad libitum* food, worms were maintained on standard NGM plates and peptone deprivation mediated DR was performed by total peptone withdrawal from standard NGM plates^22^,^55^, a condition that stops bacterial growth on the plates. Peptone deprivation not only affects bacterial growth, it also influences the osmolarity of the growth medium. We consider this included into the overall effect of peptone derived deprivation. Worms were observed daily, with nematodes transferred to new plates every day until reproduction had ceased. Worms were considered to have died if they were not moving and failed to respond to touch with a worm-pick. Any worms that died due to maternal hatching (bagging) were noted, and these data were used to investigate variation in maternal hatching in the ILs and wild isolates. As is routine for the analysis of lifespan in *C. elegans* deaths by maternal hatching were censored the analysis of lifespan.

### Maternal hatching in the RILs

To assay maternal hatching in the RILs, 40 synchronized worms per RIL were followed for 1 week after the L4 stage and the number of maternal hatchings recorded daily.

### Data analysis

Lifespan in the ILs was analyzed using Kaplan-Meier Survival curves with significance determined by log-rank analysis in Minitab® Statistical Software (Mintab Ltd., Coventry). QTL mapping of the percentage maternal hatching per RIL was done using a single marker model^34^. Data is archived in WormQTL (http://www.wormqtl.org)^56^,^57^,^58^.

## Additional Information

**How to cite this article**: Stastna, J. J. *et al.* Genotype-dependent lifespan effects in peptone deprived *Caenorhabditis elegans*. *Sci. Rep.* **5**, 16259; doi: 10.1038/srep16259 (2015).

## Supplementary Material

Supplementary Dataset 1

Supplementary Dataset 2

## Figures and Tables

**Table 1:** Lifespan and the effect of peptone deprived DR in ILs and N2.

Line	Introgression	Lifespan (days)				Maternal hatching			
		NGM	DR	effect	p-val (NGM vs DR)	NGM	DR	effect	p-val (NGM vs DR)
N2	—	17.7	22.2	23.5%	<0.0001	5.1%	0	−5.1%	NS
ewIR01	*I:* 0–2.82	15.6	22.5	41.1%	<0.0001	2.6%	0	−2.6%	NS
ewIR18	*I:* 13.28–15.10	17.7	20.8	17.1%	<0.01	5.1%	5.4%	+0.3%	NS
ewIR21	*II:* 0–4.80	14.2	13.2	−2.5%	NS	18.9%	33.3%	+14.4%	<0.01
ewIR40	*III:* 8.00–10.61	17.1	14.2	−14.9%	<0.02	18.4%	27.0%	+8.6%	<0.01
ewIR51	*IV:* 1.38–6.60	15.5	13.1	−6.4%	NS	10.0%	41.7%	+31.7%	<0.05

Shown are the mean lifespan and the percentage of worms that died by maternal hatching under *ad libitum* (NGM) and peptone deprived DR conditions, the percentage effect of DR on the trait and the significance. Introgression shows the chromosome and maximum limits (i.e. the position of the flanking N2 markers) of the portion of the CB4856 introgressed into this IL.
